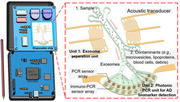# AHEADx: Revolutionizing Early Alzheimer’s Disease Diagnosis Through Blood with Integrated Acoustofluidics and Photonic PCR Technologies

**DOI:** 10.1002/alz.084184

**Published:** 2025-01-09

**Authors:** Eric V Belcea, Kim G Johnson, Andy Liu, Sheng Luo, Laurie H Sanders, Luke P Lee, Tony J Huang

**Affiliations:** ^1^ Duke University, Durham, NC USA; ^2^ Duke University School of Medicine, Durham, NC USA; ^3^ Duke University Medical Center, Durham, NC USA; ^4^ Harvard Medical School, Boston, MA USA

## Abstract

**Background:**

This study introduces the **A**utomated **H**igh‐purity **E**xosome isolation‐based **AD d**iagnostics system **(AHEADx)**. By analyzing and understanding the molecular cargo (proteins and miRNAs) carried by circulating exosomes, researchers found brain‐derived exosome (BDE) levels of P‐S396‐tau, P‐T181‐tau, and Aβ1‐42 are elevated up to 10 years prior to clinical symptoms. Currently, there is no available technology capable of simultaneously isolating and screening exosomal biomarkers for efficient and personalized precision medicine giving early AD diagnosis.

**Method:**

This NIH funded study will develop and validate AHEADx via integrated acoustofluidics (i.e., the fusion of acoustics and microfluidics) and photonic PCR on‐chip technologies that are capable of fully automated, rapid, precise exosome isolation and accurate analysis for AD diagnostics. AHEADx consists of two units: a rapid (<1 min) acoustofluidic separation unit for exosome isolation from biofluids with high yield and purity (both >90%), and a rapid (<6 min) photonic PCR unit achieving detection limits of ∼1 copy/µL for nucleic acids and ∼5 copies/µL for proteins.

Compared with state‐of‐the‐art exosome isolation and analysis technologies, AHEADx system has the following advantages:

• Automated and fast operation in a point‐of‐care, handheld system

• High‐purity (>90%), high‐quality exosome isolation for accurate biomarker detection

• High‐sensitivity (∼1 copy/µL for nucleic acids and ∼5 copies/µL for proteins) detection of a comprehensive (∼20) panel of AD biomarkers

**Result:**

To validate the potential for clinical use, we will test plasma samples from 100 AD patients and 100 healthy individuals all with known amyloid, phospho tau and total tau biomarker status measured in their CSF. The Duke University Neurology Biobank and the Duke University and University of North Carolina Alzheimer’s Disease Research Center (Duke/UNC ADRC) will provide samples.

**Conclusion:**

We predict the AHEADx platform will be capable of the simultaneous isolation and analysis of exosome‐derived biomarkers for early AD neuropathological diagnosis. The AHEADx platform’s ability to accurately detect AD biomarkers in the preclinical stages as a point of care handheld instrument could revolutionize diagnosis of AD pathology in the office setting, enhance understanding of AD progression, and significantly impact research into effective treatments.